# The development and pilot testing of an adolescent bullying intervention in Indonesia – the ROOTS Indonesia program

**DOI:** 10.1080/16549716.2019.1656905

**Published:** 2019-09-12

**Authors:** Lucy Bowes, Farida Aryani, Faridah Ohan, Rina Herlina Haryanti, Sri Winarna, Yuli Arsianto, Hening Budiyawati, Evi Widowati, Rika Saraswati, Yuliana Kristianto, Yulinda Erma Suryani, Derry Fahrizal Ulum, Emilie Minnick

**Affiliations:** a Department of Experimental Psychology, University of Oxford, Oxford, UK; b Department of Research, Yayasan Indonesia Mengabdi, Jakarta, Indonesia; c Department of Public Administration, Sebelas Maret University, Surakarta, Indonesia; d Deputy for Child Rights and Protection, Office of Women Empowerment and Child Protection Central Java, Central Java, Indonesia; e Deputy for Programme Section, Yayasan Setara, Semarang, Indonesia; f Department of Public Health, State University of Semarang, Semarang, Indonesia; g Department of Environmental and Urban Studies, Soegijapranata Catholic University, Semarang, Indonesia; h Department of Public Administration, Diponegoro University, Semarang, Indonesia; i Department of Psychology, Klaten Widya Dharma University, Klaten, Indonesia; j Child Protection Section, UNICEF Indonesia, Jakarta, Indonesia

**Keywords:** Bullying, intervention, adolescent, Indonesia, peer-led

## Abstract

Bullying has been described as one of the most tractable risk factors for poor mental health and educational outcomes, yet there is a lack of evidence-based interventions for use in low and middle-income settings. We aimed to develop and assess the feasibility of an adolescent-led school intervention for reducing bullying among adolescents in Indonesian secondary schools. The intervention was developed in iterative stages: identifying promising interventions for the local context; formative participatory action research to contextualize proposed content and delivery; and finally two pilot studies to assess feasibility and acceptability in South Sulawesi and Central Java. The resulting intervention combines two key elements: 1) a student-driven design to influence students pro-social norms and behavior, and 2) a teacher-training component designed to enhance teacher’s knowledge and self-efficacy for using positive discipline practices. In the first pilot study, we collected data from 2,075 students in a waitlist-controlled trial in four schools in South Sulawesi. The pilot study demonstrated good feasibility and acceptability of the intervention. We found reductions in bullying victimization and perpetration when using the Forms of Bullying Scale. In the second pilot study, we conducted a randomised waitlist controlled trial in eight schools in Central Java, involving a total of 5,517 students. The feasibility and acceptability were good. The quantitative findings were more mixed, with bullying perpetration and victimization increasing in both control and intervention schools. We have designed an intervention that is acceptable to various stakeholders, feasible to deliver, is designed to be scalable, and has a clear theory of change in which targeting adolescent social norms drives behavioral change. We observed mixed findings across different sites, indicating that further adaptation to context may be needed. A full-randomized controlled trial is required to examine effectiveness and cost-effectiveness of the program.

## Background

Aggressive behaviors among youth, including violence and bullying, are associated with increased risk of psychiatric disorders across the life-course, poor social functioning and educational outcomes [1,2]. The high worldwide prevalence rates and potential for harm makes addressing such behaviors a public health priority – yet one that is too often neglected. The World Health Organization considers bullying to be a major adolescent health problem, defining such acts as the intentional use of physical or psychological force against others [3]. Research predominately originates from high-income settings, yet peer violence has been found to increase risk for poor mental health and risk taking behaviors, and is associated with earlier school drop out in multiple low and middle-income settings [4].

Schools play a central role in young people’s lives that go far beyond education, and help shape social, emotional and behavioral development. Educational engagement and academic attainment are closely connected with behavioral risk factors that influence major adolescent health problems, including bullying and peer violence [5]. Prevention and health promotion activities in secondary schools have great potential for promoting engagement and student wellbeing through fostering positive and healthy school climates that reduce aggressive behaviors among youth. Systematic reviews suggest that complex, whole-school interventions are effective at reducing victimization and bullying in high-income settings [6–8]. Whole-school multi-method approaches include combinations of school-wide rules and sanctions, teacher training, classroom rules, conflict resolution training and individual counseling [8]. Whole-school approaches have been shown to be most successful at reducing bullying compared to interventions targeting only one level of the problem (e.g. compared to interventions targeting only classroom-level rules against bullying, or individual-level training such as social skills groups [8]. Whole-school interventions take a socio-ecological approach to bullying by involving bullies, victims, peers, adults, and parents and by making substantial changes to the wider school environment. In a recent meta-analysis of school-based anti-bullying programs [6], the most important components that were associated with a decrease in bullying included parent training, school conferences, videos, information for parents, improved playground supervision, disciplinary methods, work with peers, classroom rules and classroom management. In addition, the total number of components and the duration and intensity of the program for children and teachers were significantly associated with a decrease in bullying. This is in keeping with the finding of a dose-response relationship between the number of components implemented in school-based interventions and the effect on bullying [9]. More recently, anti-bullying programs such as the Kiva project have highlighted the importance of including ‘bystanders’ – children who witness bullying behaviors but who are not actively involved – into anti-bullying initiatives [10,11].

Whole-school interventions are often expensive to implement, involve substantial staff training, and have only been trialed in high-income settings, where student-staff ratios are relatively low and school resources are relatively high. A recent low-cost and simple randomized intervention, ‘Roots’ [12], implemented in 56 schools (24,191 students) in the United States used a student-driven design to influence anti-conflict social norms and behavior, reducing overall levels of school conflict by 25%. Such interventions are based upon theories that individuals observe the behavior of certain people in their community to understand what is socially normative and adjust their own behavior in response. Adolescent ‘social referents’ who have an enhanced influence over school climate or the social norms and behavioral patterns in their schools are identified, and encouraged to take a public stance against peer violence and bullying. Critically, this type of intervention is low-cost, simple to implement, and utilizes existing social network structures to maximize intervention efficacy and impact rather than involving changes to school curricula or school management time. Such an approach has a high potential for settings that lack sufficient resources to implement current anti-bullying interventions.

### The Indonesian context

The Indonesian school system is both diverse and vast. With over 50 million students and 2.6 million teachers in more than 250,000 schools, it is the third largest education system in the Asia region and the fourth largest in the world (behind only China, India and the United States) [13]. Net enrollment rates for secondary education have increased steadily in recent years, with average junior-secondary enrolment currently standing at over 80%, and gender parity has also been achieved at this level, although disparities persist across regions and socio-economic groups [14].

Indonesia continues to perform poorly relative to other countries in the region in terms of standardized international tests as indicated by the OECD Program for International Student Assessments or PISA 2015 report, where Indonesia ranked 62 out of 72 included countries [15]. Improving educational outcomes remains a huge challenge for the Government. Pupil to Teacher ratios have risen sharply at the secondary level since 2010–2011, and there are considerable variations in pupil to teacher ratios across Indonesia’s districts and regions.

Nationally representative data on bullying from the Global School Health Survey (GSHS) in 2015 suggests that over 20% of Indonesian children in grades 7–9 reported experiencing bullying in the last month [16]. Despite this, no evidence-based anti-bullying interventions have been evaluated in Indonesia. There is a strong national commitment to eliminate all forms of violence, including bullying, in schools in Indonesia, as highlighted by the Child Friendly Schools initiative, the Prevention and Elimination on Violence in Schools National Strategy, and the Elimination of Violence against Children 2016–2020 [17]. These strategies include a focus on changing the current social norms that accept, tolerate, and ignore violence, including at school settings.

We aimed to use community participatory action research methods to develop and test the feasibility and acceptability of an intervention designed to reduce bullying in secondary schools in Indonesia. We conducted two pilot studies; a non-randomised  waitlist control trial in four schools across two sites in South Sulawesi, and a randomized controlled trial across eight schools in Central Java.

## Methods

We were guided by the 2008 MRC framework for this complex intervention, and included a development phase and a feasibility and piloting phase. We began with a needs assessment, and identified the evidence base for anti-bullying interventions in Indonesia and internationally. We identified promising intervention components and tailored these to the local context using participatory action research methods. We then piloted the resulting intervention in an exploratory trial involving a total of four schools across two sites in South Sulawesi to test the feasibility of key intervention components. We then further refined the intervention based on the first pilot study, and conducted a second pilot study to test feasibility and acceptability of the modified intervention across eight schools from two sites in Central Java.

### Development phase

#### Needs assessment

Adolescents were involved in designing an online ‘U-Report’ poll with UNICEF Indonesia during a participatory workshop. UNICEF launched U-Report in Indonesia in 2015 as an, ‘innovative new platform that gives young people the chance to speak up on issues that affect their lives’. This Whatsapp, Facebook and SMS based polling mechanism enables young people aged 14–24 to share their opinions on a wide-range of topics including education, violence, health and governance. More than 2,360 adolescents responded to the poll, slightly more girls (53 per cent) than boys (47 per cent). Of the respondents, 30% said their biggest problem is bullying (F = 27% M = 32%). Of the students who reported that they had experienced bullying, 55% said that they had told no one about their experience; with reasons for not telling others including being ashamed, and considering bullying to be ‘normal’. Young people were asked what Government should do to address problems with bullying, with 64% endorsing that the Government should ‘create awareness.’

Interviews, focus group discussions and workshops, described below, were held in order to determine stakeholder’s perceptions of the need for the intervention, the current context in schools and approaches for dealing with bullying.

### Meetings with teachers, school staff and students

Guided discussions were held with school teachers, school counsellors and students from eight schools using a pre-prepared list of questions in order to gain a better understanding of the school context (including student to teacher ratio, number of counselling teachers, extra curriculum activities and current budget for these, topics covered during student orientation, parent involvement with school, whether the school was involved in the Indonesian Government’s ‘Child Friendly Schools Initiative’, school discipline practices and current practices in relation to bullying).

School head teachers, teachers, counselors and students were in agreement that there was an important need to address bullying in their schools. U-Report polls conducted in the target schools with response rates ranging from 40–80% of eligible pupils found that on average 90% of pupils who completed the surveys felt that bullying was a problem in their school. Nearly all teachers who took part in qualitative interviews reported that they had seen bullying in their school. Many stakeholders emphasized the social norms that underlined the use of violence amongst school students. Students described mocking and name-calling as normative, and often reported that they did not consider such actions to be classed as bullying, considering this to include only physical actions. Teachers also reported that actions including name-calling, shouting and hitting was a ‘normal’ thing that was part of the students’ life at school. Teachers emphasised that such behaviours were ‘natural’ since they related to broader family and social norms, and as such often expressed the view that they would be very difficult to change: ‘Calling with bad names, yelling, its just part of the culture, children were educated that way in the family, so it is normal for them’.

Students also suggested that teachers were largely unaware or did not take action in cases of verbal aggression, only becoming involved if such actions escalated to physical violence. Teachers reported that they dealt with instances of physical bullying by giving threats or sanctions, or by giving additional academic tasks to the perceived perpetrators, or giving them physical punishments, such as push-ups or being forced to clear up garbage. Teacher responses to verbal bullying included telling students who had been ‘teased’ that their friend was ‘just kidding’ and that his or her comments should not be taken seriously. No programs had been implemented that dealt specifically with bullying prior to our intervention.

### Intervention development workshop

A four-day intervention development workshop took place with various stakeholders, including students, researchers, teachers, and practice-based charities. Small group work, role-plays and guided discussions were used to discuss the topic of bullying in Indonesia, and the secondary school context more broadly. An important starting point was to discuss bullying in Indonesia, including hearing concerns about bullying from students themselves as well as examining recent national-level evidence on prevalence rates and types of bullying experiences commonly encountered. Current practice with regard to bullying in Indonesian schools was discussed, as well as identifying local resources. All stakeholders emphasised the need to involve parents in anti-bullying activities where possible, with the acknowledgement that social norms relating to violence at home were closely connected with bullying involvement at school. It was recognised that parents are typically not very engaged with schools in Indonesia, and that involving parents in the intervention would need to be ‘light touch’. The need for schools to develop an anti-bullying agreement was recognised, with the suggestion that student-input on this should be essential. The stakeholders discussed the lack of evidence on how teachers currently handled bullying, the common use of corporal punishment in schools, and the role of the school counsellor in disciplining students.

Intervention design was discussed, and the use of a waitlist design whereby all schools would eventually receive the intervention was agreed as the most appropriate for this context.

### Identifying the evidence base

Recent systematic reviews of school-based interventions targeting bullying have not identified a single successful RCT conducted in either a lower or a middle-income country [6,8,18]. There are several well-evidenced anti-bullying programs that have been found to reduce the prevalence of bulling among adolescents in high-income settings. Three notable programs with strong evidence-base of randomized controlled trials are Olweus Program, Positive Action, and KiVa, although findings among adolescents compared to children are variable, all involve core taught curriculum components, and cost upwards of £1000 to implement per school [19,20]. The context in Indonesia is one in which substantial taught components that focus on topics viewed as outside of the core academic curriculum are unlikely to be implemented – despite evidence in the West that improving social and emotional skills is associated with improved academic outcomes [5]. There is also limited teacher capacity relative to the contexts in which these interventions have been trialed, and limited financial capacity to support anti-bullying programs that require a fee to implement.

### Intervention selection

A promising low-cost anti-bullying intervention was discussed. The ROOTs intervention is a low-cost anti-bullying intervention developed and implemented in 56 schools (24,191 students) in the United States. The evaluation indicated a 25% reduction in administrative reports of conflict. The intervention uses a student-led design that is well suited to adolescents, with students supported in choosing their own anti-bullying activities to spread pro-social norms throughout the peer group. The working group discussed how activities could be easily adapted to local context. Other benefits of the intervention included the fact that is was open access with no fees attached, and that the lack of a taught curriculum fits well with the current context in Indonesia, where a great emphasis is placed on improving academic outcomes. A logic model was developed, outlining goals and objectives, the expected theory of change, and identifying key indicators of acceptability and feasibility.

The potential for scaling the intervention was discussed at all stages of intervention development and piloting.

### Overview of the intervention

The guiding principles of the ROOTS intervention are: 1) to select and work with students who are influential among their peers; 2) Students select the issues; 3) Students generate possible solutions; 4) Student initiatives are made visible to others in the student body; 5) Students use online platforms and techniques to collaborate and reach others. Approximately three weeks before the intervention, highly socially connected students are identified via student nominations. A school network survey is conducted in which students are asked to nominate up to ten students in their year whom they spent the most time with (either in person or online) in the previous few weeks. This is accompanied by a full student register for the year group in order to facilitate student responses and match nominated students. During the intervention, a trained research assistant meets with a group of highly connected students every week in order to help them identify common conflict behaviours at their school. The idea is that the intervention can then be adapted to address school-specific conflicts. Students are encouraged to become the public face of opposition to identified conflicts. For example, in the original study, connected students at each school compiled a list of conflict behaviours they could address, created hash tag slogans about those behaviours, and turned the slogans into online and physical posters. The connected students’ photos were posted next to the slogan to create an association between the ant conflict statement and each student’s identity. In another activity, connected students gave an orange wristband with the intervention logo (a tree) as a reward to students who were observed engaging in friendly or conflict-mitigating behaviours. Thus the intervention model takes the form of a ‘grassroots campaign’ in which selected students take the lead and customize the intervention to address the problems they note at their school. Notably, it lacks an educational or persuasive unit regarding adult- defined problems at their school. To maintain standardized procedures, trained facilitators follow semi-structured scripts and activity guides, which are freely available online (http://www.betsylevypaluck.com/roots-curriculum/#).

### Adapting the ROOTS intervention for use in Indonesia

**ROOTS-Indonesia: Adaption process, part 1:**

Key adaptations were made to the ROOTS program following the intervention development workshop:

1. Student ‘Agents of Change’ (i.e. highly connected students) were nominated by peers through a closed questionnaire that simply asks them to choose ten peers from *their year group* that they spend the most amount of time with inside and outside of school, face-to-face or online. This change was made due to the practicalities of large school years – it would have been impractical to give every student a copy of the full school registry, and findings from the stakeholder discussions and meetings with students suggested that the majority of students spend time with peers within their own year group.
2. Rather than implementing social network analysis, a simple tally was used to identify the most highly connected students, balancing for gender and year group, and including 10% of randomly selected students. This protocol was easier to implement and did not require sophisticated statistical skills or computer programs.
3. Stakeholders had emphasized the need to include parents within program activities, whilst recognizing that parents are typically less engaged with schools in Indonesia. A greater emphasis was therefore placed on sharing anti-bullying material with parents, including information leaflets to be given to new students and their families at orientation. Student Agents of Change were also encouraged to share their activities with their parents.
4. The ROOTS Program operates through adolescent-led activities, with highly socially connected students being selected as ‘Agents of Change’. This is somewhat unusual to the Indonesian context. It was noted that students were more shy to share opinions and ideas, and needed more support to develop activities. The number of student sessions was increased from 10 to 12, and program activities were enhanced through more detailed instructions and use of examples to reflect this. One session was delivered per week, over a duration of 12 weeks. The 12 meetings cover the following topics:

**Table:** 

Meeting number	Program component
**Meeting 1**	Program Introduction
**Meeting 2**	Identity, Group Trust, and Awareness
**Meeting 3**	Student influence and reactions to conflict
**Meeting 4**	Connect student-generated changes with behaviors
**Meeting 5**	Developing student agreement for school violence prevention
**Meeting 6**	Roleplaying positive bystander behavior
**Meeting 7**	Transitioning from Individual to School-Wide Action
**Meeting 8**	Vision for ***Roots Day***
**Meeting 9**	Going Public and Strengthening the Message
**Meeting 10**	Getting Ready for ***Roots Day***
**Meeting 11**	***Roots Day!***
**Meeting 12**	***Roots*** *Program evaluation and Action planning*
5. The original ROOTS program as trialed in the USA did not incorporate any teacher engagement. Research has indicated that school bullying is closely related both to the behavior and attitudes of school staff. Whilst corporal punishment in school settings is illegal in Indonesia, the use of harsh punishment is not uncommon, with teachers reporting in qualitative interviews that they considered physical discipline an effective tool when dealing with students involved in bullying. Furthermore, teacher attitudes towards students’ behavior often lack insight; for example our qualitative findings suggested that teachers viewed bullying as normative, and something that could not be changed. Teacher training in Indonesia is tied to the core curriculum and learning. Teachers reported that they had never received training that focused on how to identify and handle bullying.
6. We therefore combined the ROOTS program with an existing teacher-training program in ‘positive discipline’ that had already been developed and piloted in Papua, Indonesia, to build capacity of primary school teachers to avoid corporal punishment through giving them skills to implement positive discipline. Teachers received a two-day training and follow-up coaching on classroom management methods, including positive discipline, and handling students with challenging behavior and addressing bullying. The program also shares information for parents on recognizing signs of bullying through new student orientation and with the School Committee.

### Outcome measures

During the stakeholder meetings and logic model development, indicators of success were identified. These included quantitative measures of bullying, indicators of school social norms, school connectedness, and a measure of how teachers handled bullying (the Teacher Handling Bullying Questionnaire). Where possible, measures that had already been validated in Indonesia were used (e.g. the Global School Based Health Questionnaire to give prevalence estimates of bullying at baseline and follow-up). However several key measures used to evaluate our program had not been validated for use in Indonesia, including the Forms of Bullying Scale, chosen to give a better overview of the types and frequency of bullying experiences encountered, the Prescriptive and Descriptive Norms Scale used in the original ROOTS trial to measure descriptive and prescriptive social norms, and the Handling Bullying Questionnaire, chosen to measure teacher’s practices with regard to bullying. We therefore adapted and validated these questionnaires through an iterative process of researcher discussion and face validation, testing the questionnaires and discussing them item by item with an independent group of school students in South Sulawesi. Questionnaires were presented in an adolescent-friendly format, with pictures of Indonesian adolescents.

**Primary outcome measures**:

1. The Global School Based Health measure of bullying This is a single-item measure of bullying victimization prevalence, taken from the Global School Based health Survey, a self-administered, school-based survey developed by WHO in collaboration with the United Nations Children’s Fund, the United Nations Educational, Scientific and Cultural Organization, and the Joint United Nations Programme on HIV/AIDS, and with technical and financial assistance from the United States Centres for Disease Control and Prevention in Atlanta, GA. Further details of the GSHS can be obtained at http://www.who.int/chp/gshs and http://www.cdc.gov/gshs
2. The Forms of Bullying Scale. This is a 10-item scale that measures different types of bullying victimization and perpetration, including verbal, social, and physical bullying [21].

**Secondary outcome measures**:

1. Prescriptive and descriptive social norms *This is a 10-item scale that measures different types of bullying victimization and perpetration, including verbal, social, and physical bullying, developed as part of the original ROOTS study* [12].
2. The Beyond Blue School Climate Scale. *This gives a measure of student’s perceptions of the school climate, including supportive teacher relationships, sense of belonging, participative school environment and student commitment to academic values* [22].
3. Teacher Handling Bullying Questionnaire. *This gives a measure of student’s perceptions of the school climate, including supportive teacher relationships, sense of belonging, participative school environment and student commitment to academic values* [23].

### Qualitative data collection

Qualitative data collection informed our process evaluation. Eight participants (four boys and four girls) were selected by the research team for focus group discussions, including two members of ‘OSIS (Intra-School Students Organization)’, student representatives. Students were selected randomly from the school registrar, rather than chosen by teachers (a more common practice in Indonesia). Focus group discussions were guided by a set of pre-defined questions that included issues on school climate regarding bullying in school. Semi-structured interviews with a further six students were conducted individually. Data were analyzed to capture the school policies and regulations in relation to bullying.

### Facilitator training

A 4-day workshop was held to train facilitators in delivering the ROOTS intervention to schools in Indonesia. This included presentations on the intervention and logic model, the practicalities of holding student meetings (including consent and assent, timings and locations), discussion and practice in the use of facilitator skills, facilitator checklists and recording activities, and safeguarding issues. Facilitators were given a guided ‘walk through’ of two ROOTS sessions, and then took turns delivering the other sessions to the group, with feedback sessions after each delivery.

### Pilot testing of ROOTS-Indonesia in South Sulawesi: fidelity monitoring and documentation of adaptation

Four secondary-high schools were selected in South Sulawesi; two in Makassar, and two in Gowa. School locations were selected with consultation of the Local Education Office to include both rural and urban contexts as well as to ensure availability of referral services in the case that serious cases were identified. Although the intention had been to randomize schools, this was not done due to school scheduling difficulties. Quantitative measures were taken at baseline and at 5-month follow-up. The total number of students included in the intervention across South Sulawesi was 2,075 (see Table 1 for sociodemographic characteristics of students at baseline).

Attendance rates for the 12 facilitated students meetings varied, ranging from just 8 to 40 with an average of 29 students (73% of total group). Girls were typically more likely to attend than boys.

### Process evaluation

A facilitator checklist was developed to aid organization and implementation of Agents of Change meetings. In addition, a diary was developed prior to program commencement to record key details regarding the Agents of Change meetings including number of pupils attended, gender balance, comments on the program, changes needed to be made etc. Facilitators filled in the diary after each session at each site.

### Feasibility of the intervention

The research team set clear targets for intervention activities in our log frame. We aimed for each intervention school to have a Agents of Change group of 40 students with at least 60% of high social referents included. Each intervention school identified highly socially connected students through a student blind voting system and included over 60% of these students in the Agents of Change group. It was clear that this was an unusual method of selecting students, and some teachers initially expressed dissatisfaction that they could not select suitable students themselves. It was noted that selected students included gang members and individuals who themselves typically engaged in bullying. However, teachers reported seeing a change in the behavior of such students:

“I was surprised to see that our students became more confident after the training. They tried to act in ways that were friendly towards others, and remind others to also act in this way”

Several teachers reported that though initially skeptical, they felt that the inclusion of students who would not typically be selected by teachers was beneficial.

The average number of students per session was 27 – or 67.5% of the eligible Agents of Change group. Agents of Change students took part in a range of activities, including creating anti-bullying hash tags and organizing a ‘ROOTS day’ for all students in the school to attend. They also helped to set up online U-Report surveys, which were completed by approximately half of the total student body at each school, highlighting the reach of the program.

The positive discipline and bullying prevention training reached an average 91% of teachers at each school (range 75–100%).

### Quantitative data collection in South Sulawesi

We measured bullying in two ways: A simple, single-item measure from the Global School Based Health Surveys in order to be able to report the total prevalence rates of bullying as a percentage, and the more detailed Forms of Bullying Scale. The latter has greater reliability and validity, and allows us to examine rates of different types of bullying, as well as enhanced statistical power for analyses.

### Prevalence rates of bullying at baseline

At baseline, 57.5% of the students in South Sulawesi reported being bullied at least once or twice in the last 30 days. The most prevalent types of bullying were verbal (e.g. nasty teasing and name calling) and social (stories told about them, false rumors spread to damage social standing). In common with many other studies, girls were slightly more likely to report social and relational victimization, whilst boys were more likely to report threats and physical violence.

### Students’ perceptions of student behavior at baseline

Students also reported that they often saw other students engaged in aggressive behavior – 19% of students in South Sulawesi reported that they saw students threatening, hitting, or punching others on a daily basis. Students reported rarely seeing other students defending someone if people were trying to hurt them, with 27% of students reporting that they ‘never’ saw this type of prosocial behaviour, and 25% also reporting that they never saw other students reporting conflicts to a teacher or parent.

### Effects of the pilot intervention on bullying prevalence in South Sulawesi

In South Sulawesi, reports of bullying victimization increased in the intervention group when using the single item Global School Based Health (GSHS) measure (Intervention schools: 55.4% to 57.9%, at follow-up, control schools 59.7% baseline; 52.0% follow-up, Table 2). However, when we used the more detailed Forms of Bullying Scale, we found that reports of bullying victimization reduced significantly more in intervention schools compared to control schools. Specifically, there was an 11% reduction in mean victimization scores in intervention schools, giving a Cohen’s D effect size of 0.39, a small-medium effect size. Reports of bullying perpetration also decreased from M 4.00 (SD 4.61) at baseline to M 2.86 (SD 4.01) at outcome in the intervention group (a 17% reduction in mean rates) compared to M 4.12 (SD 4.52) to M 2.96 (SD 3.86) in the control group.

**Table 1.** Sociodemographic characteristics of students at baseline (N = 1,901).

Category	Intervention	Control	Total
Age (M, SD)	13.02 (0.93)	13.00 (0.76)	13.01 (0.85)
Sex (%)			
Male	46.9	44.7	45.8
Female	53.1	55.3	54.2
School year (%)			
VII	53.9	51.3	52.7
VIII	46.1	48.7	47.3

**Table 2.** Student-reported bullying prevalence in South Sulawesi at baseline and follow-up, according to intervention group.

	Intervention		Control	
Measure	Baseline	Follow up	Baseline	Follow up
GSHS (%)	55.4	57.9	59.7	52.0
FBS (Total victimization)	7.61 (6.51)	6.07 (5.93)	7.67 (6.38)	6.51 (6.32)
Verbal victimization	2.32 (2.07)	2.04 (1.95)	2.36 (2.05)	2.17 (2.00)
Threats – victimization	1.15 (1.57)	0.86 (1.41)	1.20 (1.74)	0.93 (1.59)
Physical – victimization	1.21 (1.61)	0.95 (1.44)	1.34 (1.66)	0.97 (1.46)
Relational – victimization	1.64 (1.89)	1.16 (1.57)	1.56 (1.73)	1.34 (1.72)
Social – victimization	1.35 (1.73)	1.12 (1.60)	1.29 (1.65)	1.19 (1.68)
FBS (Total perpetration)	4.00 (4.61)	2.86 (4.01)	4.21 (4.52)	2.96 (3.86)
Verbal perpetration	1.75 (1.91)	1.25 (1.54)	1.92 (1.92)	1.44 (1.71)
Threats – perpetration	0.65 (1.21)	0.51 (1.09)	0.60 (1.15)	0.46 (0.96)
Physical – perpetration	0.44 (1.10)	0.36 (0.94)	0.47 (1.12)	0.33 (0.97)
Relational – perpetration	0.64 (1.23)	0.41 (0.98)	0.60 (1.09)	0.40 (0.88)
Social – perpetration	0.57 (1.19)	0.38 (0.95)	0.56 (1.09)	0.40 (0.95)
School climate (total)	15.83 (6.48)	14.84 (6.92)	17.79 (6.10)	15.96 (6.48)
Descriptive norms	22.32 (9.09)	20.71 (8.77)	24.14 (9.61)	21.25 (8.72)
Prescriptive norms	28.07 (12.13)	26.50 (13.78)	30.55 (13.00)	26.13 (12.89)

Qualitative analysis supported a drop in bullying perpetration at the South Sulawesi site. One teacher noted that including students who were actively engaged in bullying perpetration in the Agent of Change group had a positive impact:

“Initially, I thought this behavior could not be changed. But then I saw one of the agents of change was the “trouble maker” in our school. After the training, he seemed to be able to control himself. He even told his fellows to stop annoying others”

There was no significant change in adolescent’s perception of student behaviours and norms. There was also no overall change in positive school climate as measured by the Beyond Blue School Climate Scale.

We measured teachers’ awareness and attitudes in response to bullying using the Handling Bullying Questionnaire administered to teachers at baseline, midline and follow up. We found that teachers reported that they were less likely to ignore the bullying at follow-up than at baseline, and were more likely to report working with the bully and the victim.

### Stage 3: pilot testing of ROOTS-Indonesia in Central Java

Eight secondary-high schools were selected in Central Java; four in Klaten, and four in Semarang. Once again, school locations were selected with consultation of the Local Education Office to include both rural and urban contexts as well as to ensure availability of referral services in the case that serious cases were identified. Quantitative measures were taken at baseline and at 7-month follow-up. Schools were randomized to intervention or wait list control within each of the two sites. The intervention delivered involved both the student-led component and the teacher-training component. The total number of students included in the intervention across Central Java was 5,517 (see Table 3 for sociodemographic characteristics of students at baseline). Teacher training was delivered to 89–100% of teachers per school.

### Quantitative data collection in Central Java

#### Prevalence rates of bullying at baseline

At baseline, 65.4% of the students in Central Java reported being bullied at least once or twice in the last 30 days. Whilst the prevalence of bullying victimization varied per school (from 49.4% to 79.0%), the overall rates of bullying were relatively high. The most prevalent types of bullying were verbal (e.g. nasty teasing and name calling) and social (stories told about them, false rumors spread to damage social standing). In common with many other studies, girls were slightly more likely to report social and relational victimization, whilst boys were more likely to report threats and physical violence.

#### Students’ perceptions of student behavior at baseline

Students also reported that they often saw other students engaged in aggressive behavior – for example, 47% of students in Central Java reported that they saw students threatening, hitting, or punching others on a daily basis.

#### Effects of the pilot intervention on bullying prevalence in Central Java

In Central Java, self-reported rates of victimization as measured by the single-item GSHS measure were higher at baseline in the intervention groups (68.0%) compared to the control group (63.2%). Rates of bullying increased in both the intervention and control group, by 10.8% and 6.8% respectively (See Table 4). Similarly, an increase in bullying victimization across both intervention and control groups was seen when using the FBS. Reports of bullying perpetration also increased for both the intervention and the control groups (a 7% increase in mean in the intervention group compared to 4% increase in mean in the control group).

**Table 3.** Sociodemographic characteristics of students at baseline (N = 5,308).

Category	Intervention	Control	Total
Age (M, SD)	13.3 (1.14)	13.2 (1.01)	13.26 (1.07)
Sex (%)			
Male	53.5	50.5	51.9
Female	46.5	49.5	48.1
School year (%)			
VII	39.8	32.7	36.0
VIII	32.2	34.1	33.2
IX	27.9	33.2	30.8

**Table 4.** Student-reported bullying prevalence in Central Java at baseline and follow-up, according to intervention group.

	Intervention		Control	
Measure	Baseline	Follow up	Baseline	Follow up
GSHS (%)	68.0	78.8	63.2	70.0
FBS (Total victimization)	7.77 (6.74)	9.13 (7.08)	7.48 (6.18)	8.52 (6.71)
Verbal victimization	2.94 (2.39)	3.20 (2.27)	2.73 (2.13)	2.97 (2.19)
Threats – victimization	0.87 (1.48)	1.16 (1.71)	0.88 (1.41)	1.00 (1.54)
Physical – victimization	1.20 (1.62)	1.51 (1.76)	1.04 (1.44)	1.40 (1.69)
Relational – victimization	1.26 (1.70)	1.50 (1.79)	1.36 (1.73)	1.48 (1.79)
Social – victimization	1.50 (1.79)	1.76 (1.88)	1.48 (1.70)	1.68 (1.77)
FBS (Total perpetration)	4.00 (4.28)	4.58 (4.92)	3.85 (4.24)	4.19 (4.11)
Verbal perpetration	2.32 (2.11)	2.40 (2.06)	2.13 (1.99)	2.32 (1.97)
Threats – perpetration	0.43 (0.98)	0.55 (1.14)	0.45 (1.00)	0.43 (0.93)
Physical – perpetration	0.41 (0.96)	0.56 (1.18)	0.43 (0.98)	0.51 (1.05)
Relational – perpetration	0.37 (0.90)	0.50 (1.08)	0.40 (0.90)	0.43 (0.93)
Social – perpetration	0.46 (1.00)	0.57 (1.12)	0.45 (0.95)	0.49 (0.98)
School climate (total)	17.63 (7.48)	18.10 (7.36)	16.74 (7.36)	17.89 (7.30)
Descriptive norms	31.87 (9.46)	34.27 (8.87)	32.21 (9.19)	34.13 (8.42)
Prescriptive norms	24.00 (11.24)	23.46 (11.50)	25.66 (12.25)	25.57 (12.31)

There was a small increase in positive school climate in the intervention condition as measured by the Beyond Blue School Climate Scale. In intervention schools, fewer teachers endorsed the view that physical discipline was needed to discipline children (17% in intervention schools versus 23% in control schools), and were slightly more likely to say that rather than hitting the child, it was better to explain his or her mistakes (95% versus 92%) when examining results from the Handling Bullying Questionnaire.

#### Facilitators to intervention delivery

Building a strong relationship with school staff clearly facilitated intervention delivery. Teacher support for Agents of Change selection and activities is essential to ensuring that the intervention is both feasible and acceptable. Key factors in schools where the intervention appeared most acceptable were the engagement and support of the headmaster, understanding and support from other teachers, and regular communication between the facilitators and school staff. Engaging and fun facilitators were also crucial; whilst we included lengthy facilitator training as part of the intervention, it was noted that most interactions between adults and adolescents were hierarchical and involved teaching rather than facilitation. Using experienced facilitators who are able to build good rapport with young people appeared to be a crucial element of intervention success.

Students had the opportunity to take part in anonymous U-Report polls during the intervention. Over 90% of students polled reported that they had experienced bullying, with the vast majority reporting that they did not tell a teacher about the bullying. Providing students with an anonymous platform to share experiences in this way may encourage more student disclosure and help school staff better understand the issue of bullying in their schools. For schools with limited internet access, ‘change’ boxes where students can post anonymous notes may serve a similar purpose.

#### Barriers to intervention activities

No school opted out of the intervention. Timings of student exams in the second semester presented a challenge to 3^rd^ year students being included as Agents of Change at one of the sites. This barrier was addressed by beginning the intervention in the first semester instead of the second.

Facilitators noted in their diaries that transport was one issue for reduced attendance at Agents of Change meetings:

‘One of the reasons why we couldn't have meetings after the school schedule was that many students go home together since they lived far from the school. Their friends couldn't wait, and there wasn't any vehicle or public transportation to the students' homes.''

Identifying a time for the Agents of Change student meetings remains a challenge; teachers typically did not want students to miss classes in order to take part in the intervention. Holding meetings outside of core school hours however increased the likelihood of student transportation issues. Identifying time during school extracurricular sessions proved helpful in this context, but it is likely that meeting timings need to remain flexible, with adaptations made for individual school contexts. Increased awareness of the impact of bullying on educational attainment and student wellbeing may also help to convince teachers that such activities should play a more central role on school curriculums.

Scheduling conflicts presented a strong barrier to student attendance, increasing the likelihood of student drop out from the Agents of Change group. Hesitance to participate in mixed-gender and mixed-age group activities was noted, particularly in initial meetings, and irregular attendance to meetings was also observed. These barriers were addressed by highlighting the important role of the Agent of Change students during meetings, encouraging ‘icebreaking’ activities, and where timings of meetings were an issue, altering the times where possible. The attendance of group members typically increased gradually as students became more comfortable with each other, resulting in enhanced diversity over time. In particular, the schoolwide ROOTS day was well-attended by students. Building a strong relationship with teachers and giving clear information to parents about the benefits of being involved in intervention activities would facilitate attendance.

A lack of teacher knowledge and awareness on child development and the causes and consequences of bullying remains a challenge. Teachers typically only actively intervened when bullying escalated to physical violence, and considered many aspects of bullying to be normative. To overcome this barrier, we included a 4-day training workshop for teachers on positive discipline. It was noted however that prior to this, teachers had received almost no training on child wellbeing, and that a 4-day training programme may not be enough to tackle widespread and entrenched views concerning bullying and violence. UNICEF has developed a comprehensive programme for teachers on positive discipline and classroom management, which includes training as well as ongoing coaching support to change entrenched norms around the acceptability of violence as well as norms around how children are viewed in Indonesian culture.

Negligible participation of parents in school governance and day-to-day proceedings remains a challenge. A ‘light touch’ approach was taken, whereby Agent of Change students were encouraged to discuss their activities with their parents. Information about bullying was also included in material given out to parents at school orientation.

## Discussion

The evidence base for the design and sustainable delivery of anti-bullying interventions in low and middle-income countries is poor. We have described the development and piloting stages of the ROOTS-Indonesia program across four sites in Indonesia, home to the fourth largest education system in the world. Our aim was to develop a scalable program that targeted adolescent social norms that sustain and promote bullying and aggression. Our methodology was informed by the approach recommended by the MRC framework for the design and evaluation of complex interventions [24], involving evidence synthesis, formative research, and two pilot studies.

Evidence from high-income settings suggests that bullying is tractable, and that whole-school multicomponent interventions can successfully reduce the prevalence rates of bullying. We add to this literature in several ways. First, we use participatory delivery methods, including facilitating selected students in developing their own anti-bullying messages, and in sharing them with others through their media of choice, and through whole school activities such as U-report polls that offer a platform to enable students to share their views in an anonymous way. Second, we highlight the importance of including teacher training within an adolescent-led program in order to strengthen teachers understanding of the causes and consequences of bullying. Third, we highlight the importance of adolescent social norms with regard to bullying and violence. Whilst these must be viewed in the context of broader social norms with relation to the use of violence and aggression, we provide evidence that adolescent social norms are malleable, and that by targeting such norms, we may change students interactions. We emphasize that engagement and support of headmasters, and involvement of teachers in supporting intervention activities are key requirements for acceptability and feasibility. Our study fits with other evidence that indicates that the participation of teachers, family, community members and students in the design and implementation of the program, and the use of participatory, active learning techniques where young people have the opportunity to develop skills, identify their own issues, and have an active voice in combatting them, are key drivers of the acceptability and feasibility of a school-based intervention [25,26].

Our study was underpowered to detect significant effects. Nevertheless, we found that reports of bullying perpetration and victimization decreased in South Sulawesi when assessed using the detailed Forms of Bullying Scale. Qualitative findings supported the feasibility and acceptability of the intervention in South Sulawesi. In Central Java however, our findings were more mixed, with reports of victimization and perpetration increasing in both control and intervention conditions. Qualitative analyses were largely more positive, with facilitators and some teachers noting very positive effects in the behavior of the student Agents of Change. There are several factors that may explain the discrepancy in findings. It is possible that awareness of bullying increased during the program, leading to more pupils identifying that they had experienced bullying. Simply asking students about bullying can lead to an increased awareness, which may explain the finding that bullying increased for the control group as well. Alternatively, it is possible that local events outside of the intervention may have led to a real increase in bullying prevalence during this time for both intervention and control groups. We also cannot rule out that our program may have led to increases in bullying in one site. Given our mixed findings, and to ensure that our program does not lead to increases in bullying, further adaptation and evaluation is warranted. We suggest that future adaptations seek to screen out students with high levels of antisocial behavior from the selected Agents of Change students to minimize the risk of any peer deviancy effects the intervention could inadvertently lead to. Other psychosocial trials have indicated that whilst peer-led intervention programs can be very effective [27], they can also lead to increases in antisocial behavior where children with high levels of conduct problems are brought together as one of the program components [28].

### Cost of the program

Key costs are for the program are: payment of facilitators, snacks provided to Agents of Change, promotional material for activities of Agents of Change (including banners for Roots Day, pins etc.); teacher training costs. The cost effectiveness of the intervention and potential cost savings if scaled up will be assessed in detail in the next phase through a costing study supported by UNICEF.

### Sustainability of the program

The sustainability of the program is dependent on having good facilitators who can communicate effectively both with students and with school staff and parents. Program effectiveness, like other whole-school programs focused on systemic behavior change, is likely to take more than one year to ‘bed in’ (typically 2–3 school academic years), so it will be important for schools involved to have a sustainable method both of selecting student agents of change, and in terms of access to facilitators. UNICEF is currently working with district and province government to ensure that these components are included in the government scale up plans. In South Sulawesi, government and schools have opted to embed the program with an existing extracurricular program called OSIS (Organisasi Siswa Intra Sekolah), while in both provinces facilitators will continue to be recruited from the existing program of the government called Forum Anak or Child Forum.

## Conclusion

Bullying is a major risk factor for poor educational, health and social outcomes. Despite evidence from high-income countries that whole-school interventions are effective at reducing prevalence rates of bullying, there is a lack of evidence-based, sustainable programs for low-resource settings. Our study addressed the feasibility and acceptability of delivering a novel peer-led intervention, ROOTS-Indonesia, for reducing peer violence and bullying among adolescents in a lower-middle income setting. We have designed an intervention that is acceptable to various stakeholders, feasible to deliver, is designed to be scalable, and has a clear theory of change in which targeting adolescent social norms drives behavioral change. Our anti-bullying program was aimed at underlying social norms that promote bullying and violence among high school students. Our program is unique in that these social norms are being targeted through participatory methods. Aside from teacher training, there are no formative lessons on bullying, but rather key, socially-connected students are developing and delivering their own anti-bullying messages throughout their school networks. We pilot tested the intervention across two diverse states in Indonesia, in order to examine the potential generalizability of the program to the varying socio-cultural settings of schools in Indonesia. We found important differences across sites in the effectiveness of our intervention, emphasizing the need to understand and adapt to differences in local context. In the next phase of the program, UNICEF will support the government through the process of replication to other schools using their own budget, with technical support and capacity building from UNICEF. Future government-led implementation will be evaluated through UNICEF support.

Our program is unique in several ways. First, we use participatory delivery methods, including facilitating selected students in developing their own anti-bullying messages, and in sharing them with others through their media of choice, and through whole school activities such as U-report polls that offer a platform to enable students to share their views in an anonymous way. Second, we highlight the importance of including teacher training within an adolescent-led program in order to strengthen teachers understanding of the causes and consequences of bullying. Third, we highlight the importance of adolescent social norms with regard to bullying and violence. Whilst these must be viewed in the context of broader social norms with relation to the use of violence and aggression, we provide evidence that adolescent social norms are malleable, and that by targeting such norms, we may change students interactions. We emphasize that engagement and support of headmasters, and involvement of teachers in supporting intervention activities are key requirements for acceptability and feasibility. Our study fits with other evidence that indicates that the participation of teachers, family, community members and students in the design and implementation of the program, and the use of participatory, active learning techniques where young people have the opportunity to develop skills, identify their own issues, and have an active voice in combatting them, are key drivers of the acceptability and feasibility of a school-based intervention.

## Paper context

Bullying is a risk factor for poor mental health and educational outcomes. In Indonesia, over 20% of children in grades 7–9 reported experiencing bullying in the last month. We developed an adolescent-led school intervention for reducing bullying in Indonesia. It included two key elements: 1) a student-driven design to influence anti-conflict social norms and behavior among students; 2) a teacher-training component to enhance teacher’s knowledge and self-efficacy for using positive discipline practices.
